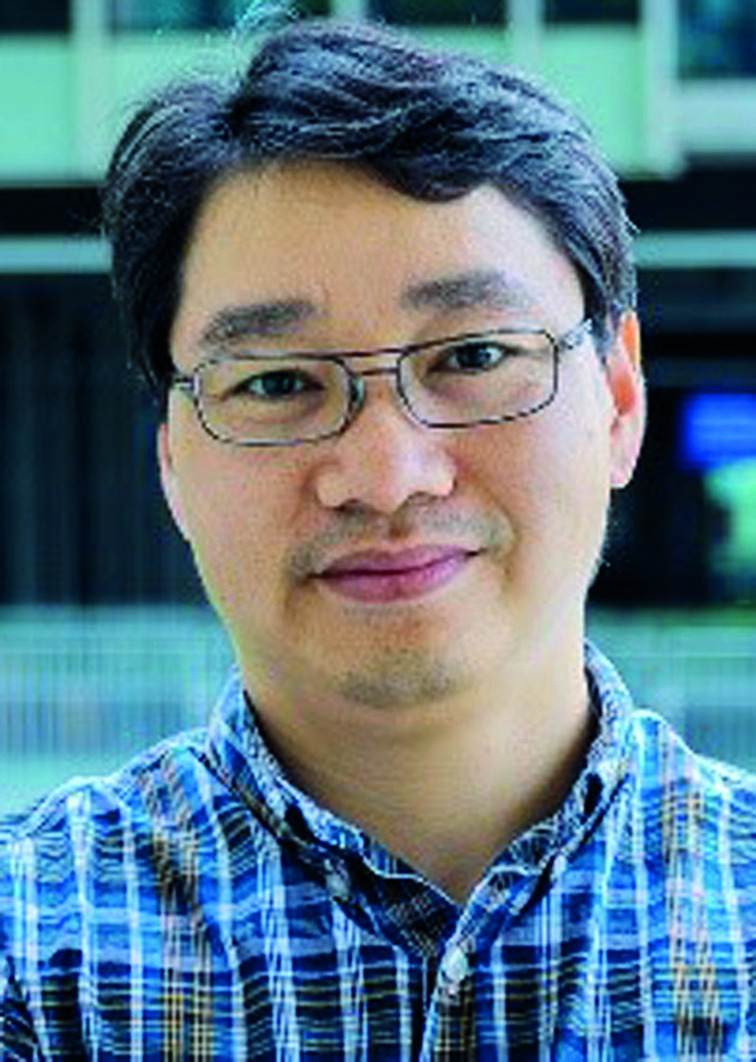# 3D Printing MXene‐Based Electrodes for Supercapacitors

**DOI:** 10.1002/asia.202400568

**Published:** 2024-10-18

**Authors:** Xudong Jiang, Juan Bai, Binodhya Wijerathne, Qianqin Zhou, Fan Zhang, Ting Liao, Ziqi Sun

**Affiliations:** ^1^ School of Chemistry and Physics Queensland University of Technology 2 George Street Brisbane, QLD 4000 Australia; ^2^ School of Mechanical Medical and Process Engineering Queensland University of Technology 2 George Street Brisbane, QLD 4000 Australia; ^3^ Centre for Materials Science Queensland University of Technology 2 George Street Brisbane, QLD 4000 Australia

**Keywords:** 3D printing, MXene, Supercapacitor, Energy storage

## Abstract

3D printing, as an advanced and promising strategy for processing electrode for energy storage devices, such as supercapacitors and batteries, has garnered considerable interest in recent decades. The interest in 3D printed electrodes stems from its exceptional performance and manufacturing features, including customized sizes and shapes and the layer‐by‐layer processing principle, etc., especially integrating with MXene which allows the manufacturing of electrodes from different raw materials and possessing desired electrochemical properties. Herculean challenges, such as material compatibility of the printing inks, nondurable interfacial or bulk mechanical strength of the printed electrodes, and sometimes the low capacitance, lead to inferior electrochemical performance and hinder the practical applications of this promising technology. In this review, we firstly summarize the representative 3D printing methods, then, review the MXene‐based 3D printing electrodes made from different materials, and last, provide electrochemical performance of 3D printing MXene‐based electrodes for supercapacitors. Furthermore, based on a summary on the recent progress, an outlook on these promising electrodes for sustainable energy devices is provided. We anticipate that this review could provide some insights into overcoming the challenges and achieving more remarkable electrochemical performance of 3D printing supercapacitor electrodes and offer perspectives in the future for emerging energy devices.

## Graphical abstract

**Testing nobility**: This review integrates the advances in 3D printing of MXene‐based electrodes, which offers flexible and customized processing strategies, and the advantages of MXenes electrodes with unparallel electrochemical and mechanical properties for supercapacitors. It is expected to provide some insights into the design and manufacturing of electrodes for supercapacitors.

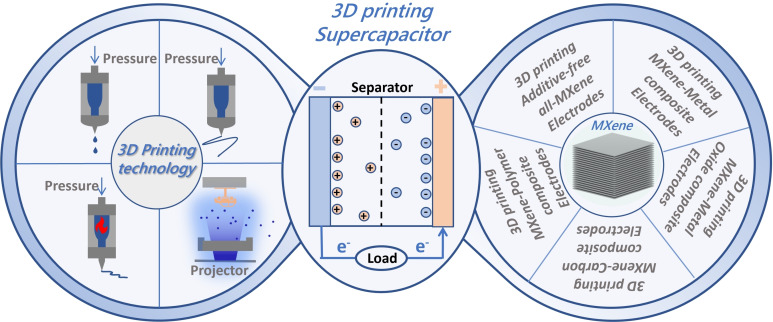

## 1 Introduction

To cope with the increasing demand in energy and the worsening environment, there has been undergoing a revolutionary change in energy supplies and storages over the past few decades, especially the utilization of electrochemical energy.[^1^, ^2^, ^3^, ^4^, ^5^, ^6^] As a trend, a plenty of energy devices are emerged, such as rechargeable batteries,[^7^, ^8^, ^9^] electrocatalyzing fuel generations,[^10^, ^11^] high‐capacity supercapacitors,[^12^, ^13^, ^14^] and so on. As a promising type of energy storage devices, supercapacitors have attracted wide attention, due to their excellent charge and discharge rate performance, high power density, long cyclic lifespan, and superior stability and safety.[^15^, ^16^] Supercapacitors are a sort of electronic devices for storing and releasing significant amounts of electrical energy via double‐layer capacitance, electrode adsorption, or pseudocapacitance.^[17]^ Compare to current rechargeable batteries, supercapacitors exhibit higher power density and longer cycle life. Therefore, supercapacitors are very promising in transportation, electronic medical devices, renewable energy storages, industrial productions, aerospace technologies, and smart electronic devices.^[18]^ The manufacturing and assembly of electrodes with precisely controlled geometric shapes and structures govern the energy and power densities of supercapacitors.^[19]^ While the noted advantages of supercapacitors, to meet the requirements in real‐world applications, significant endeavours in further enhancing the electrochemical performance via proper material and architecture optimizations are yet needed to be heavily committed.

Traditional electrode manufacturing often leads to material loss and waste. In contrast, 3D printing technology offers a more precise and flexible manufacturing option, which can significantly reduce the losses and minimize the consumption of high‐value raw materials.^[20]^ Furthermore, 3D printing technology can fabricate size tailorable and complex structures for energy storage electrodes, such as microporous and multilayer structures, thereby improve the supercapacitor charge‐discharge efficiency.[^21^, ^22^] Diverse 3D printable materials, ranging from polymers, metals, ceramics or metal oxides, to carbon materials, are available for fabricating supercapacitor electrodes. The variations of materials properties and functionalities, however, demand meticulous consideration and selection of an appropriate 3D printing technology, otherwise some practical issues, such as poor printing quality, excessive materials consumption, and low production efficiency, etc., would appear.

In the selection of a printing technology, the physical and chemical properties of the ink and active materials and the desired functionality should be carefully considered. For polymer‐based materials, extrusions of liquid‐state oligomer or melted polymers integrating with a light‐induced processing, such as the combination of Fusion Deposition Modelling (FDM) together with Stereolithography or Digital Light Processing (DLP), are usually employed to fabricate solid supercapacitor electrodes. For the printing of this type of material, the thermoplastic properties, the unsaturated bonds, and the epoxy groups of the polymers are very critical for high‐quality printing.[^23^, ^24^] For printing metal oxide and carbon‐based materials, extrusion printing of inks, such as Direct Ink Writing (DIW) and Ink Jetting Printing (IJP), has been commonly used for electrode fabrication. In the printing of this class of materials, a suitable rheological property is the key to ensure smooth extrusion and stable structure construction.[^25^, ^26^, ^27^, ^28^] To enhance the structural robustness, selective laser sintering (SLS) has been developed for constructing stable metal oxides‐based electrodes.[^29^, ^30^, ^31^] Therefore, for the successful 3D printing of electrodes, the selection of an appropriate 3D printing technology against the material properties and demands for maintaining sufficient electrode integrity is the first step.

MXenes are a group of two‐dimensional (2D) transition metal carbides and nitrides and have been recognized as a promising class of energy materials, due to their substantially high specific surface area and flexibility. MXene materials are named based on their chemical formula of M_*n+1*_X_*n*_T_*x*_, where M represents a transition metal, X is a non‐metal element, such as carbon and nitrogen, n is the number of layers, and T_*x*_ is the functional groups. The commonly used MXenes in energy devices include Ti_3_C_2_T_*x*_, Ti_2_CT_*x*_, V_2_CT_*x*_, Mo_2_CT_*x*_, and Nb_2_CT_*x*_.^[32]^ Especially, MXenes have been widely employed in supercapacitors.^[33]^ There are mainly two types of supercapacitors, according to the distinctive energy storage mechanisms ‐ electrochemical double layer capacitors (EDLCs) and pseudocapacitors. For the application in EDLCs, the layered structure of MXene provides a large specific surface area to increase the charge storage capacity. Additionally, MXene allows ions to quickly transfer between layers, which significantly enhances the charge‐discharge rate and response speed. In pseudocapacitors, the multilayered structure of MXenes can also provide a large number of reactive sites for Faradaic reactions, which contributes to high charge storage capacity and enhanced charge transfer efficiency.^[34]^

Some limitations, however, exist in MXene materials. The complex preparation process of MXene involving the use of hazardous acids, such as HF and HCl^[35]^ and the existence of unexpected surface groups,^[36]^ thereby have impacted the scalability and sustainability of the production and application of these materials. Sharing one commonest issue with other 2D materials, the exfoliated MXene sheets intend to undergo restacking attracted by the van der Waals forces.^[37]^ Once MXene sheets restack, the effective surface area available for ion adsorption is reduced, leading to a lower overall capacitance. For the practical application of MXene‐based energy materials, new synthesis strategies are needed to enhance the material productivity and environmental sustainability and to reduce the reliance on hazardous acid solutions during the synthesis procedures. Heterostructuring of MXene with other materials and introducing surface‐active functional groups can reduce restacking and enhance the structural stability and durability.[^38^, ^39^, ^40^] This strategy could ensure the preparation of uniform MXene‐based inks for 3D‐printing. Furthermore, proper nanoengineering, including size or thickness modulation, solvent selection, and surface group modification, could also be a tangible strategy for solving the restacking issue, especially for preparing high concentration inks for printing high load electrodes.[^41^, ^42^, ^43^]

**Figure 1:**
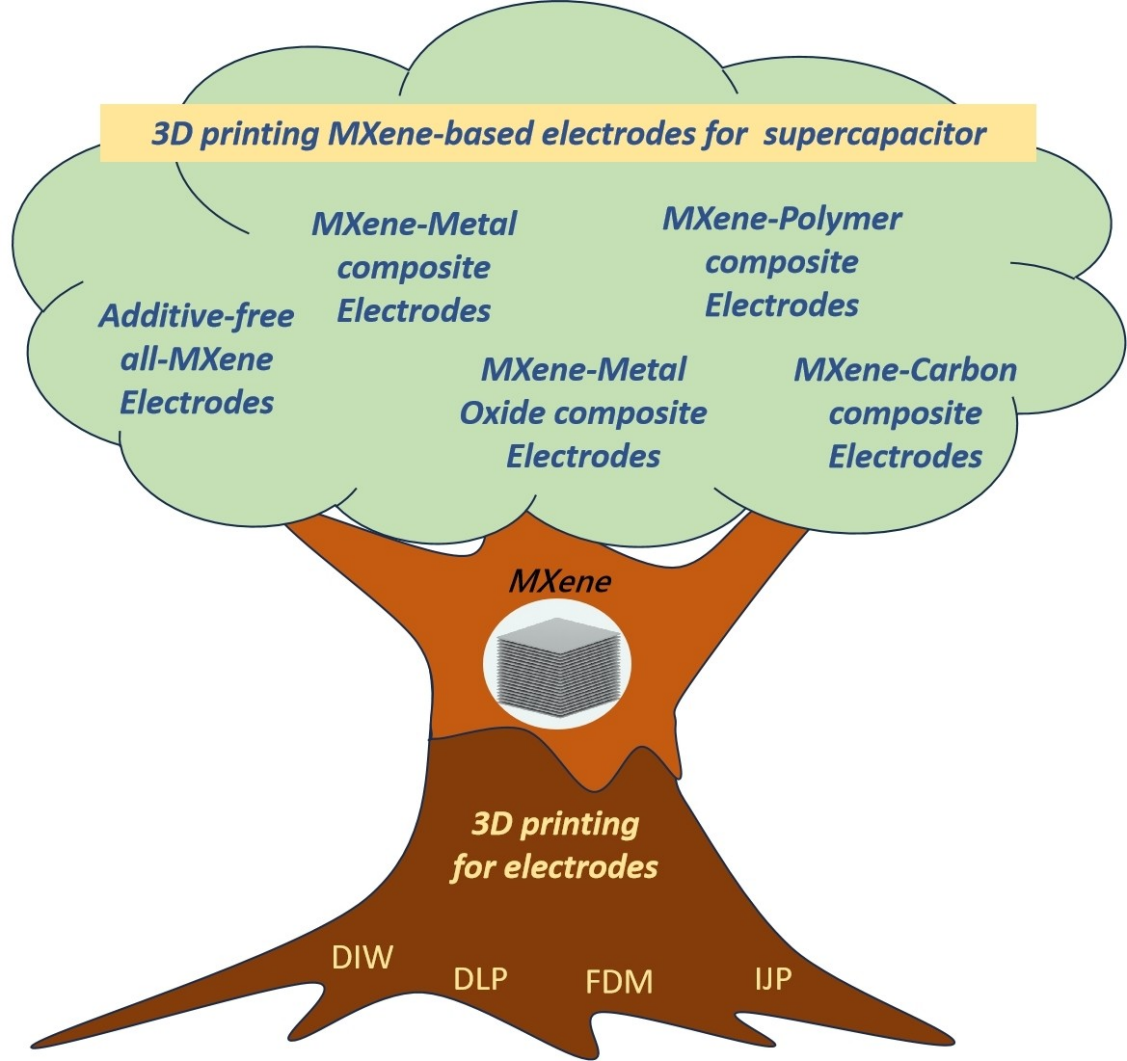
Schematic illustration of materials and techniques for 3D printing of MXene‐based electrodes for supercapacitors. The MXene‐based electrode materials include (i) additive‐free all‐MXene electrodes, (ii) MXene‐polymer composite electrodes, (iii) MXene‐carbon composite electrodes, and (iv) MXene‐metal oxide composite electrodes. 3D printing techniques used for electrode fabrication include Direct Ink Writing (DIW), Digital Light Processing (DLP), Fused Deposition Modelling (FDM), and Ink Jetting Printing (IJP).

In this review, we summarize four majorly available 3D printing technologies for electrode preparations and then review the recent progress in 3D‐printing MXene‐based electrodes for supercapacitors. Based on the critical review on the advancements in various 3D printing methods suitable for MXene‐based electrode materials and the electrochemical performance of the printed electrodes, some insights into solving the challenges of 3D printing with MXene‐based materials are offered. In general, utilizing 3D printing technology to process MXene‐based composite electrodes is akin to tree roots delivering nutrients to the crown. The synergy between the 3D printing and MXenes can produce a variety of high‐performance electrodes for energy devices. With the continuous efforts of numerous researchers, the research on 3D printed MXene‐based electrodes will flourish and bear abundant fruit (Figure 1).

## 2 3D Printing Technologies Applied in Electrodes Fabrication

**Figure 2:**
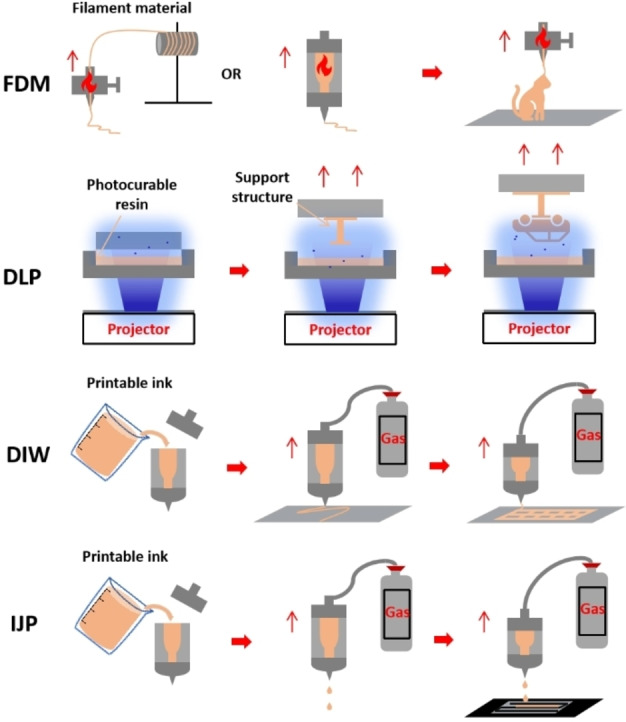
Major 3D printing methods employed for electrochemistry device manufacturing: Fusion Deposition Modelling (FDM), Digital Light Processing (DLP), Direct Ink Writing (DIW), and Ink Jetting Printing (IJP).

3D printing technology has been widely utilized in the manufacture of supercapacitors^[44]^ and has been regarded as the hallmark of the third industrial revolution, due to its ability in meeting personalized customization demands. Typically, objects constructed layer‐by‐layer along the Z‐axis could be precise controlled over dimensions and geometry.^[45]^ A typical 3D printing process involves four key steps, i. e., 3D structure modelling, slicing processing, 3D printing, and post‐processing. To date, various 3D printing techniques have been garnering significant attention for customizing supercapacitors. Notably, Fusion Deposition Modelling (FDM), Digital Light Processing (DLP), Direct Ink Writing (DIW), and Ink Jetting Printing (IJP) methods are particularly favoured by researchers for electrochemistry device manufacturing. Leveraging the unique features of these 3D printing techniques and the innovative microstructures of materials, electrodes with targeted performances and properties can be reliably developed.^[46]^ Therefore, each of those 3D printing methods involves distinctive processing strategies, as shown in Figure 2. Compared with traditional methods, 3D printing offers a more promising approach to tailor the geometry, porosity, and morphology of the conductive electrodes in energy devices, thereby enhancing the specific energy density and the power density.^[47]^ This section explores the available 3D printing technologies in electrode fabrication for energy storage devices.

### 2.1 Fusion Deposition Modelling (FDM) 3D Printing

FDM is a widely applied printing method and often utilized for the production of relatively large size components. Compared with other 3D printing technologies, FDM is a very facile and simple method. In terms of starting materials, there are two types of FDM 3D printing technologies.^[48]^ The one is FDM 3D printer with filament extrusion with the feeding of filament‐shape printing materials. The other is FDM with pellet‐shape starting materials. During printing, both the filament and the pellets need to pass the printing nozzle for heating and melting and then extruding to build up the designed 3D structures. Typically, FDM is more suitable for the rapid printing of polymer‐based materials, which have lower melting points and could facilitate a rapid and smooth melting for the extrusion process. Despite this method not being efficient for printing materials with high melting points, the strong mechanical properties of the used polymer materials still endow them with a significant potential in the preparation of electrodes.

**Figure 3:**
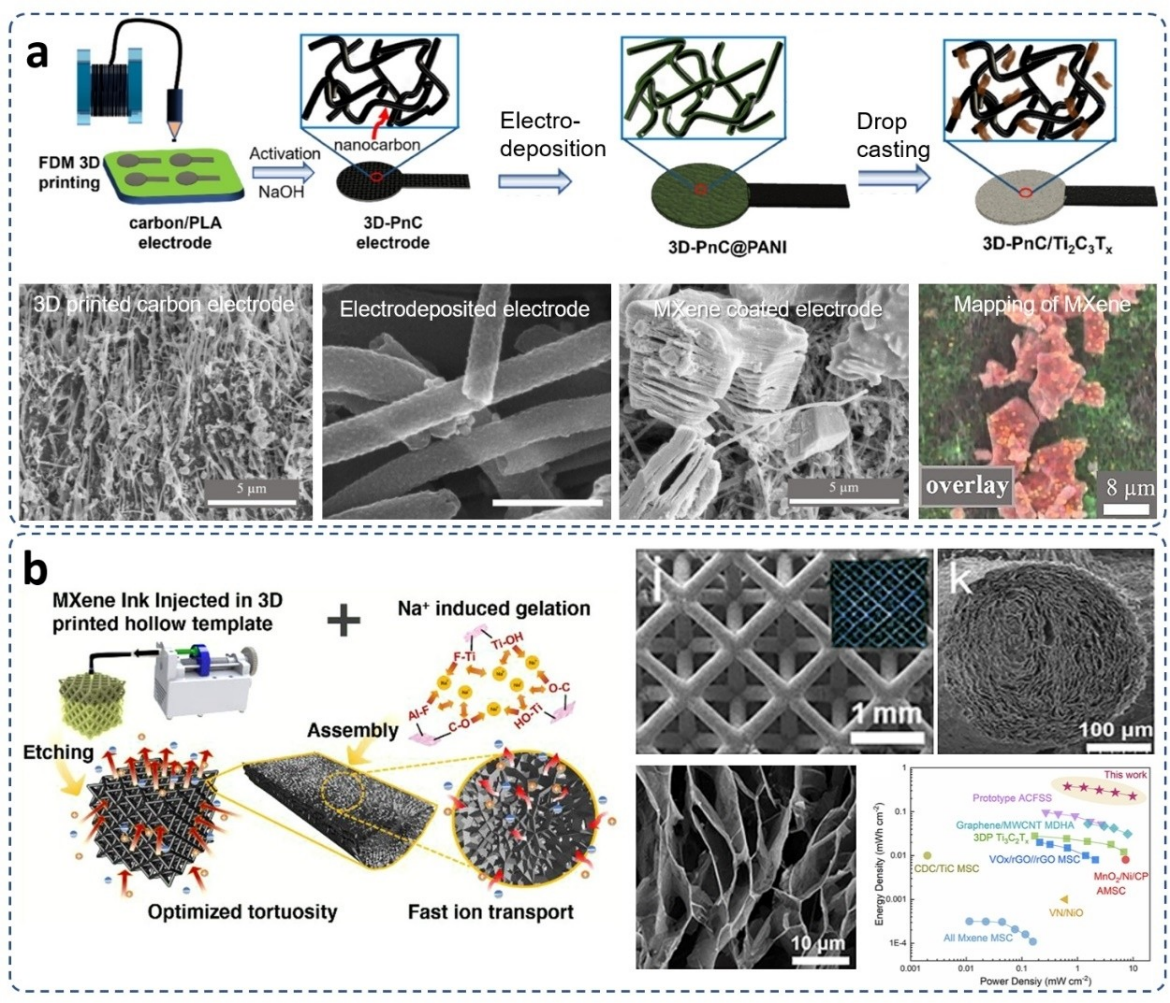
MXene‐based supercapacitor electrodes fabricated via typical 3D printing techniques. (a) MXene/nano carbon electrodes fabricated via a Fused Deposition Modelling (FDM) method. The SEM images represent the microstructures of the printed electrodes after stepwise treatments. Reproduced under terms of the CC‐BY license of Taylor & Francis Group.^[51]^ Copyright 2024. (b) The schematic illustration of the preparation of 3D printed all‐MXene electrodes via a Digital Light Processing (DLP) method, and the corresponding microstructures and electrochemical performance. Reproduced from Ref. [56] with permission (2022) from American Chemical Society.

To date, a significant number of polymer‐based electrodes have been developed with satisfactory electrochemical properties by utilizing the FDM 3D printing method. Hu et al. fabricated two types of 3D printed electrodes by using thermoplastic polyurethane (TPU)/Lithium iron phosphate (LiFePO_4_) and TPU/lithium titanate (Li_4_Ti_5_O_12_), respectively. Both electrodes achieved nearly 100 % capacitance retention after 300 cycles.^[49]^ Similarly, by utilizing the FDM 3D printing technology, a polyethylene (PE) electrode structure was firstly prepared and subsequently electrochemically deposited a nickel‐cobalt layered double hydroxide onto the surface (NiCo‐LDH@3D‐PE) as the electrodes of supercapacitor. This 1.6 mm‐thick NiCo‐LDH@3D‐PE electrode demonstrated a high mass loading of 15.3 mg cm^−2^ and exhibited a capacitance of 25.9 F cm^−2^, equivalent to 1690 F g^−1^, at a current density of 10 mA cm^−2^ and 93 % capacitance retention after 10,000 cycles.^[50]^ Besides the printing of polymer‐based electrodes, FDM 3D printing can also be used for fabricating carbon‐based electrodes. By using a FDM 3D printing method, Mappoli et al. fabricated a nano‐carbon electrode with a uniform MXene coating (Figure 3a), which demonstrated a slow capacitance decay for enhanced conductivity and cycling stability and retention of 76 % capacitance after 20,000 cycles.^[51]^

Although the low printing precision and quality, FDM 3D printing still remains as one of the major 3D processing technologies for the manufacturing of polymer‐based materials. In the fabrication of energy devices, this method can still be considered as a reliable 3D printing technique that is capable of producing electrodes with high cycling stability.

### 2.2 Digital Light Processing (DLP) 3D Printing

DLP 3D printing is a technology that employs the interaction between the light emitting diode (LED) light at 405 nm wavelength and photo‐curable resins to print structures in a layer‐by‐layer manner.^[52]^ This method can achieve the printing of three‐dimensional objects at an accuracy of 50 μm,^[53]^ which is much higher than the FDM 3D printing method. Furthermore, the resin used in this processing is at a fluidic slurry state, which circumvents the need of raw material pre‐treatment as that in the FDM 3D printing. In the process, a 3D modelling of the structure should be submitted into the 3D system of the DLP printer for slicing into layers. After that, the 405 nm LED light is emitted by a projector according to the pattern of the slicing model of each layer onto the photopolymerizable resin to perform a layer‐by‐layer gradual curing process. Via this approach, complex architectures with high resolutions could be fabricated.

Via the utilization of the DLP 3D printing technology, a porous graphene foam supercapacitor electrode achieved a capacitance of 0.81 F cm^−3^ at a high current density of 75 mA cm^−3^ and a remarkable mechanical compression strength of 1.4 MPa. After 16000 compression fatigue cycles, the electrode performed a little plastic deformation.^[54]^ A light‐curable hydrogel‐based octet‐truss electrode fabricated by the DLP 3D printing technology also performed a high capacitance of 19.53 mF cm^−3^ at 0.1 mA cm^−3^ at a temperature as low as −30 °C. Under high deformation, this microelectrode performed an excellent electrical property with capacitance retention of 96.8 % at room temperature.^[55]^ In another case, Yang et al. built an all‐MXene electrode for supercapacitor by DLP 3D printing and sodium cation‐induced self‐gelation to achieve impressive porous architectures, which reached a high areal capacitance of 7.5 F cm^−2^ at a scan rate of 0.5 mA cm^−1^ (Figure 3b).^[56]^

One of the obvious advantages is the DLP 3D printing can construct structures at a high resolution. It is very helpful in the fabrication of electrode with complex shapes, porous architectures, or high surface areas, which are critical factors to reach high capacitance of supercapacitors. It is surely that there are some challenges in this processing technology. In manufacturing electrodes at large‐scale or large‐size, structure deformation or collapse could happen, due to a long curing time is required for the photopolymerization of the liquid resins. For this shortage, the application of DLP printing technology in electrode manufacturing yet remains in lab research.

### 2.3 Direct Ink Writing (DIW) 3D Printing

DIW 3D printing is based on an extrusion process realized by external pressure.^[57]^ In this 3D processing technique, the viscosity of the printing inks is very critical, which determines whether the structures can be successfully fabricated. Inks with unsatisfactory viscosity usually cause either the blockage of the print nozzles or the collapse of the structure before effective curing, and thus lead to poor quality of the products. Therefore, the viscosity of inks needs to be carefully adjusted with the assistance of additives.

Even if the existence of the disadvantages, the DIW 3D printing technology holds significant practical potentials for its simple setup and working principle and has been widely employed in the fabrication of conductive electrodes in a variety of shapes. This method can be used for all materials that can be prepared into highly fluidic inks and has been successfully applied to electrode manufacturing. For instance, Zhao et al. fabricated a reduced graphene oxide (rGO)/KCu_7_S_4_ electrode via this 3D printing method, which reached remarkable electrical chemistry performance, i. e., the areal capacitance and volumetric capacitance reached 7.33 F cm^−2^ and 27.7 F cm^−3^ at 0.5 A g^−1^, respectively. In this case, the KCu_7_S_4_ provides effective shuttle of potassium ions and enhanced the electrical conductivity, and the lamellar rGO supports the mechanical strength of the 3D architectures.[^58^, ^59^]

**Figure 4:**
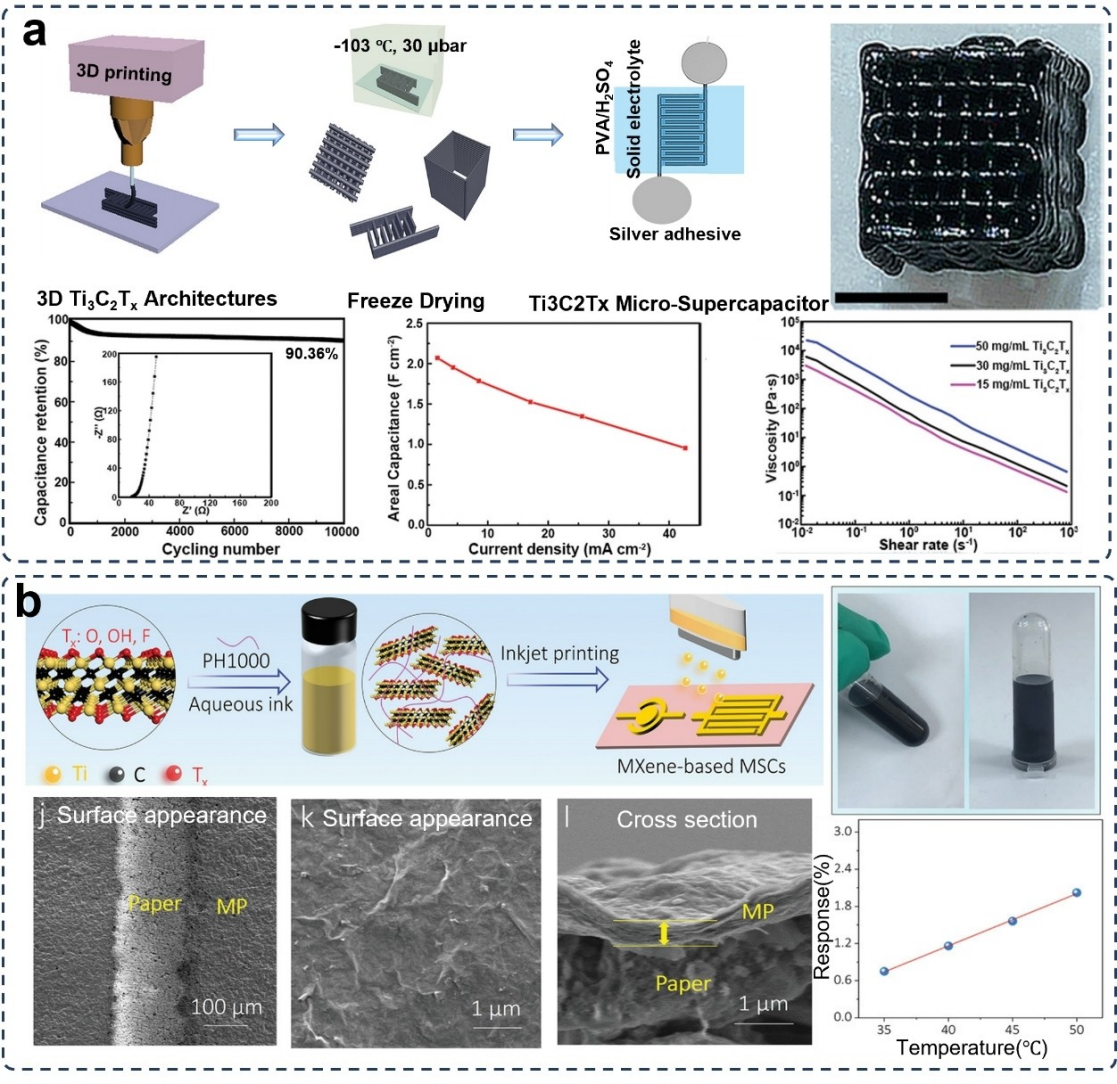
MXene‐based supercapacitor electrodes fabricated via typical 3D printing techniques. (a) illustration of 3D printing of additive‐free MXene electrodes via a Direct ink Writing method, and the corresponding microstructures. Reproduced from Ref. [60] with permission (2019) from Wiley‐VCH. (b) Procedure illustration of 3D printing of MXene/polymer electrodes via a ink jetting printing (IJP) method and the corresponding photograph of the prepared inks and microstructures of the printed electrodes. Reproduced from Ref. [66] with permission (2021) from Wiley‐VCH.

As the use of additives could affect the conductivity of the printing ink, the addition of conductive additives or the development of non‐additive inks is becoming another approach. Yang et al. printed an additive‐free MXene‐based electrode for supercapacitors (Figure 4a). Aqueous ink composed of atomically thin (1–3 nm) Ti_3_C_2_T_*x*_ with ideal viscoelastic properties was used for DIW 3D printing. The 3D printed electrode reached a high areal capacitance of 2.1 F cm^−2^ at 1.7 mA cm^−2^ and an 90 % capacitance retention after 10,000 cycles.^[60]^

In this printing process, the ink is extruded and stacked by the nozzle under external forces. Hence, the flowability of the inks becomes a primary factor affecting the quality of printed products. In some cases, the tiny particles in the inks tend to aggregate at the nozzle and eventually result in clogging. While the dilution of the inks can enhance the fluidity for avoiding clogging, the low storage modulus results in the deformation and poor mechanical properties of the printed structures. For the practical application of this technology, the development of stable and highly dispersed inks with high solid content and satisfactory rheological properties is the key step.

### 2.4 Ink Jetting Printing (IJP) 3D Printing

IJP printing is an extrusion‐based non‐contact printing methodology, wherein the ink droplets are precisely extruded from a nozzle and sequentially deposited onto the substrate. This process facilitates the rapid formation of a predetermined pattern, leveraging the precision and efficiency inherent of this technology.^[61]^ According to the distinctive mechanisms, IJP printing technique can be categorized into two different types: (i) continuous IJP printing and (ii) drop‐on‐demand IJP printing. Continuous printing is realized by extruding printing ink continuously. In this process, the ink drops for printing are deposited onto the substrate, while part of the ink drops is collected and recycled by applying an extra electrical field. Compared with the continuous printing, the drop‐on‐demand printing allows individual drops being ejected on desired location and fabricates models in a more economical way, through the control of the applied air pressure.^[62]^ Moreover, if combining with heating, the drop‐on‐demand printing can build architectures on the substrate from high‐temperature melts. Due to the shared similarity in working principles with DIW, the IJP printing method also provides a wide range of material selections and customized manufacturing as the DIW. However, the IPJ method has higher printing accuracy for its better control of a stable air pressure to extrude drops uniformly.

Morshed et al. fabricated an ultra‐thin supercapacitor composed of silver (Ag) and dielectric materials by using the IJP technology.^[63]^ The total thickness of the ultra‐thin supercapacitor was 23 μm, and the capacitance with three repeated layers of a 7 μm Ag layer and a 1 μm dielectric layer reached 412.5 pF mm^−3^. By taking the advantages of the IJP printing technique, a freestanding paper electrode was printed from a homogenous ink by dispersing graphene hydrogel‐polyaniline nanocomposite in a water‐ethanol mixture for supercapacitor. This flexible all‐solid‐state electrode for a symmetric supercapacitor achieved an energy density of 24.02 Wh kg^−1^ at a power density of 400.33 W kg^−1^ and capacity retention of 85.6 % after 5,000 cycles.^[64]^

As mentioned above, the restacking issue of MXene is severe, especially in aqueous inks with high solid concentration. To solve this issue, besides the introduction of electrostatically repelling surface functional groups, the addition of steric reagents is also a very promising solution.^[65]^ Ma et al. successfully fabricated an aqueous MXene/poly(3,4‐ethylenedioxythiophene):poly(styrenesulfonic acid) (PH1000) hybrid ink, whose preparation procedure can be found in Figure 4b. It is worth noting that the PH1000 polymer is of great aqueous solution processibility and the chain structures can be easily to adhere to the surface of MXene to provide a steric effect for avoiding self‐stacking of the MXene nanosheets. The directly ink jetting printed micro‐supercapacitor delivered a volumetric capacitance of 754 F cm^−3^ and an energy density of 9.4 mWh cm^−3^.^[66]^ It is more noteworthy that this inkjet printing technique offers a straightforward and scalable potential for producing freestanding graphene‐based nanohybrid electrodes, whose property and geometry can be precisely tailored to reach an optimal performance.

It is surely some challenges existing in this technology. Apart from the nozzle clogging similar to the DIW, multiple factors, such as the rheological properties of the inks and the wettability, the adhesion, and the structure of the substrates, have imposed constraints on the achievable geometries. While the existence of challenges, this technique has a great potential in its practical application in manufacturing thin film electrodes, micro‐electronic devices, and ultra‐thin supercapacitors.

## 3 MXene‐Based Electrodes Fabricated by 3D Printing

To date, various materials, such as polymer‐based, carbon‐based, and metal oxide‐based materials, have been employed for manufacturing of 3D‐printed conductive electrodes for energy devices.^[67]^ The diverse materials possessing distinct chemical and physical properties not only endow the printed electrodes with miscellaneous electrochemical performances, but also bring extra challenges in the selection of 3D printing methods and the determination of manufacturing parameters. For example, metal‐oxide based materials, such as ruthenium oxide, exhibit high specific capacitance, good conductivity, and excellent stability and cyclability,^[68]^ but usually require pre‐treatment and post‐treatment processes in their 3D printing manufacturing.^[69]^ In some cases, multiple materials are used to obtain desired conductivity and performance. Issues of uniformity of materials in the mixture lead to problems of ink extrusion and deposition.

Since the discovery by Naguib et al. in 2011,^[70]^ MXenes have been deemed as an emerging group of electrode materials for achieving high capacitance and energy density.^[71]^ Significant progress has been attained in manufacturing MXene‐based electrode by using 3D printing technologies. It has been reported that the volumetric capacitance and energy density of MXene‐based electrodes for supercapacitors/micro‐supercapacitors surpassed many other active materials.[^72^, ^73^] The high affinity towards water and other organic solvents and the high compatibility with other additives also facilitate the preparation of aqueous inks and precursors for extrusion‐based 3D printing technologies with their desirable viscosity and shear behaviours. Based on the components in the inks, the 3D‐printed MXene‐based electrodes for supercapacitors are classified into four groups: additive‐free all‐MXene electrodes, MXene‐polymer based electrodes, MXene‐carbon based electrodes, and MXene‐metal oxide‐based electrodes. Due to the unique material properties for each group, different 3D printing technologies have been applied to the manufacturing of MXene‐based electrodes. In this section, recent progress for each type of MXene‐based electrodes is reviewed and some possible solutions in addressing the challenges of 3D printed MXene‐based electrodes are offered.

### 3.1 3D Printing Additive‐Free All‐MXene Electrodes

MXenes are a typical class of 2D materials, which possesses excellent hydrophilicity, robust mechanical properties, and also outstanding electrochemical properties.^[74]^ These salient properties make this class of materials to be excellent candidates for strong and high‐performance electrodes for emerging energy devices, including supercapacitors. Furthermore, the desirable rheological and shear‐thinning behaviors of 2D MXenes enable the uniform preparation of printing inks without the addition of other additives, which help maintain the unique properties of MXenes without the interferences from the additives.^[75]^ Therefore, the additive‐free all‐MXene electrodes exhibit strong potential in the application for supercapacitors.

As 3D printing provides straightforward, direct, and cost‐effective construction of electrodes with customized sizes, structures, and shapes. It has been demonstrated that MXene materials can be prepared into high‐concentration inks with desired rheological properties for extrusion printing of diverse structures and thicknesses on various substrates.^[57]^ To achieve a high concentration in printing inks while maintaining uniform dispersion, multiple approaches have been proposed. Yuan et al. prepared a uniform MXene solution and then regulated the concentration by using centrifugation and magnetic stirring for printing. Quasi‐solid‐state symmetrical micro‐supercapacitors prepared through direct ink writing printing presented not only excellent electrochemical performance with a capacitance of 2.337 F cm^−2^ at 2 mV s^−1^ and capacitance retention of 93.1 % after 10000 cycles, but also robust mechanical properties enabling the maintenance of its original electrochemical performance under bending deformations.^[76]^ In another report, the concentration of the additive‐free MXene (Ti_3_C_2_T_*x*_) 3D printing ink was regulated by the addition amount of the organic solvent. The 3D printed electrode displayed a stabile capacitance retention of 97 % up to 14,000 cycles.^[73]^ The preparation of MXene with desired loads can also be achieved through solvent evaporation, which, however, may take very long evaporation time, especially at room temperature and lead to the issues of segregated sedimentation and restacking.^[77]^

**Figure 5:**
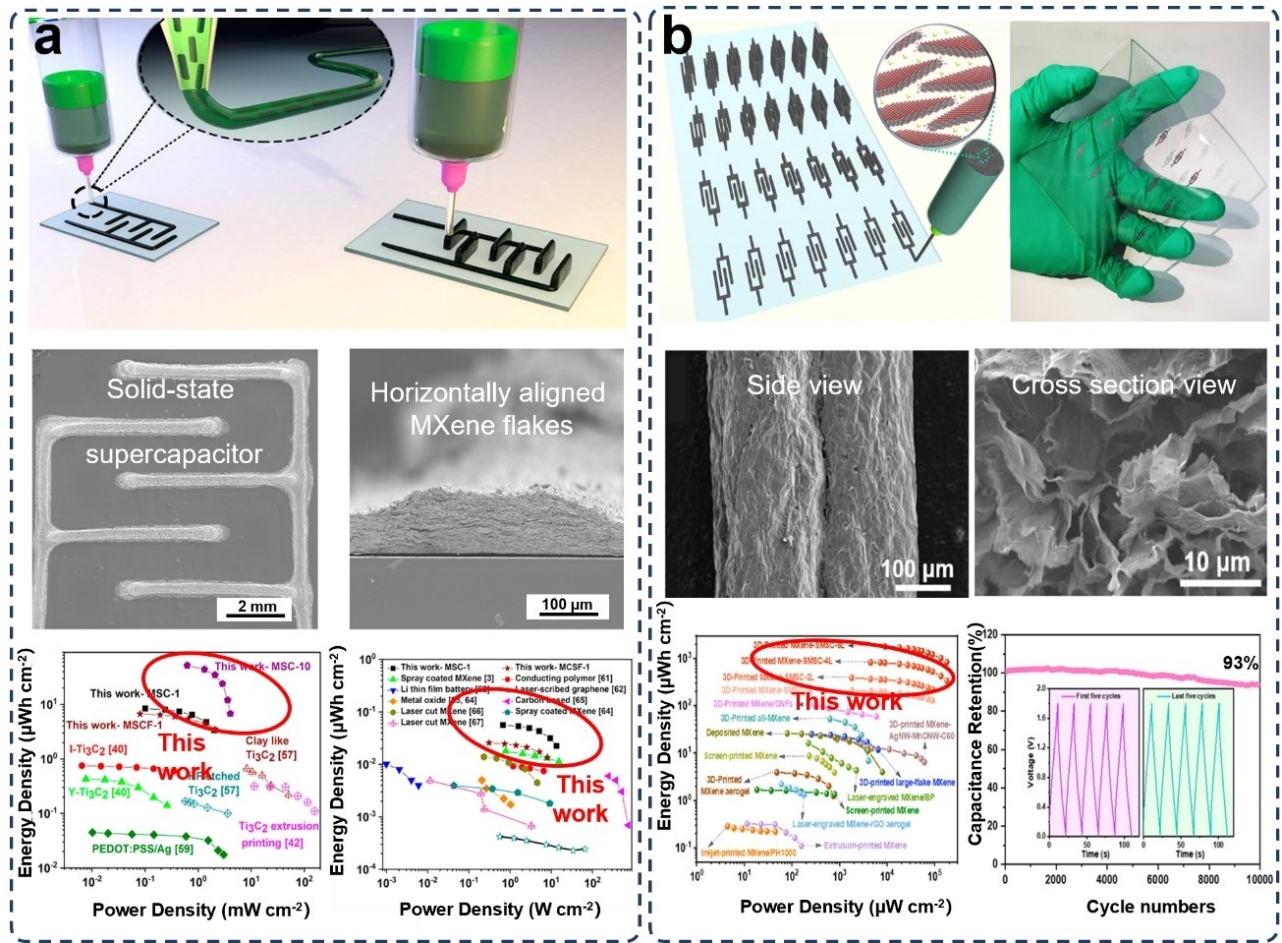
Additive‐free/all‐MXene electrodes for supercapacitors prepared via 3D printing. (a) DIW 3D printing of additive‐free MXene and the corresponding microstructure for supercapacitors, showing outperforming energy density and power density to the electrodes of other MXenes other active materials. Reproduced from Ref. [79] with permission (2020) from American Chemical Society. (b) DIW 3D printing of micro‐supercapacitors composed of all‐MXene electrodes and a water‐in‐lithium bromide (LiBr) gel electrolyte for high energy density and stabile capacitance retention. The SEM images represent the side view and cross section view of the micro‐supercapacitor. Reproduced under terms of the CC‐BY license of Wiley‐VCH.^[82]^ Copyright 2024.

It is clear that the regulating the concentration of all‐MXene ink is still fraught and full of considerable challenges. Orangi et al. designed a 3D printing method for electrodes assembly.^[78]^ In this method, polymer beads made from cross‐linked polyacrylate‐based copolymer were used to absorb the solvent for obtaining high‐concentration aqueous MXene inks with satisfactory rheological performance but avoiding the introduction of other additives or long‐time evaporation process.^[79]^ By taking advantage of this ink, a DIW 3D printed solid‐state supercapacitor was obtained, as shown in Figure 5a. The 3D printed all‐MXene supercapacitor showed an exceptional areal capacitance of 1,035 mF cm^−2^ at a scan rate of 2 mV s^−1^ in a H_2_SO_4_/PVA gel electrolyte. This 3D printed electrode outperformed the MXene electrodes prepared by other methods. For example, the supercapacitor with MXene electrodes fabricated via a template‐assisted vacuum drying method exhibited a lower areal capacitance of 12.5 mF cm^−2^ at a scan rate of 5 mV s^−1^ in the same H_2_SO_4_/PVA gel electrolyte.^[80]^ By using a similar vacuum drying assembly method, the supercapacitor made of MXene/carboxymethyl cellulose electrodes performed a capacitor of 113.13 mF cm^−2^ at an approximate scan rate of 1.8 mV s^−1^.^[81]^ The 3D printed MXene‐based electrodes for supercapacitors also can be intergraded with distinct types of electrolytes. To reach a higher voltage window and better environmental durability, the supercapacitors composed of 3D printed additive‐free MXene electrodes and a water‐in‐lithium bromide (LiBr) gel electrolyte (Figure 5b) demonstrated an exceptional high voltage window of 1.8 V and an ultra‐high areal energy density of 1772 μWh cm^−2^ at a temperature as low as −40 °C and an ultra‐high areal capacitance of 3901 mF cm^−2^ at 5 mV s^−1^.^[82]^

Therefore, it has demonstrated the high feasibility to prepare 3D printable inks with requested viscosity and shear behaviors without the addition of additives. The 3D printing technologies help provide a reliable pathway for the preparation of thin film, solid‐state, and micro‐sized electrodes for high‐capacity devices for extreme environmental applications. All‐MXene materials can be prepared into high‐concentration 3D printing inks without the interference from the additives, exhibiting excellent structural integrity, durable environmental stability, and promising electrochemical performance.

By employing proper synthesis method and surface modifications, impressive electrochemical performances have been reached by 3D‐printed All‐MXene supercapacitor electrodes. The issue of restacking of the closely packed 2D MXenes during the preparation of inks resulted by the van der Waals forces, however, still remains. The restacking is also a challenging problem in balancing the concentration of MXene in 3D printing of all‐MXene electrodes, where high concentration leads to severe restacking, while lower concentration might result in insufficient density of the electrode materials. For MXene suspensions, the concentration of surface functional groups and the composition govern the colloidal stability, rheological characteristics, and film‐forming tendencies. Therefore, the introduce of strongly charged functional groups to modify the surface chemistry of MXenes can be an effective approach for preparing highly dispersed and stable suspensions from sufficient electrostatic repellence. As alternatives, the use of new technologies or the combination with other processes, such as vacuum filtration and microscopic 3D printing technology, to allow the printing of low concentration or low‐dose inks, in which the interlayer contact can be minimized, into high‐load electrode. This could also be a promising direction for printing high‐load all‐MXene electrodes.

### 3.2 3D Printing MXene‐Metal Composite Electrodes

It has been widely reported that composites can provide better electrochemistry performances compared with the sole MXene material by taking the advantage of the complementary properties of the constitutional materials.^[83]^ The metallic compounds, including metals, alloys, and intermetallic compounds, usually have better electronic conductivity and have been added into the MXene‐based electrodes for improving the electronic properties of the electrodes. Due to the distinct physical characteristics and electrochemical properties exhibited by various types of metallic materials, carefully selection on the appropriate materials is very critical to meet the specific needs for modulating the performance of the electrodes.

**Figure 6:**
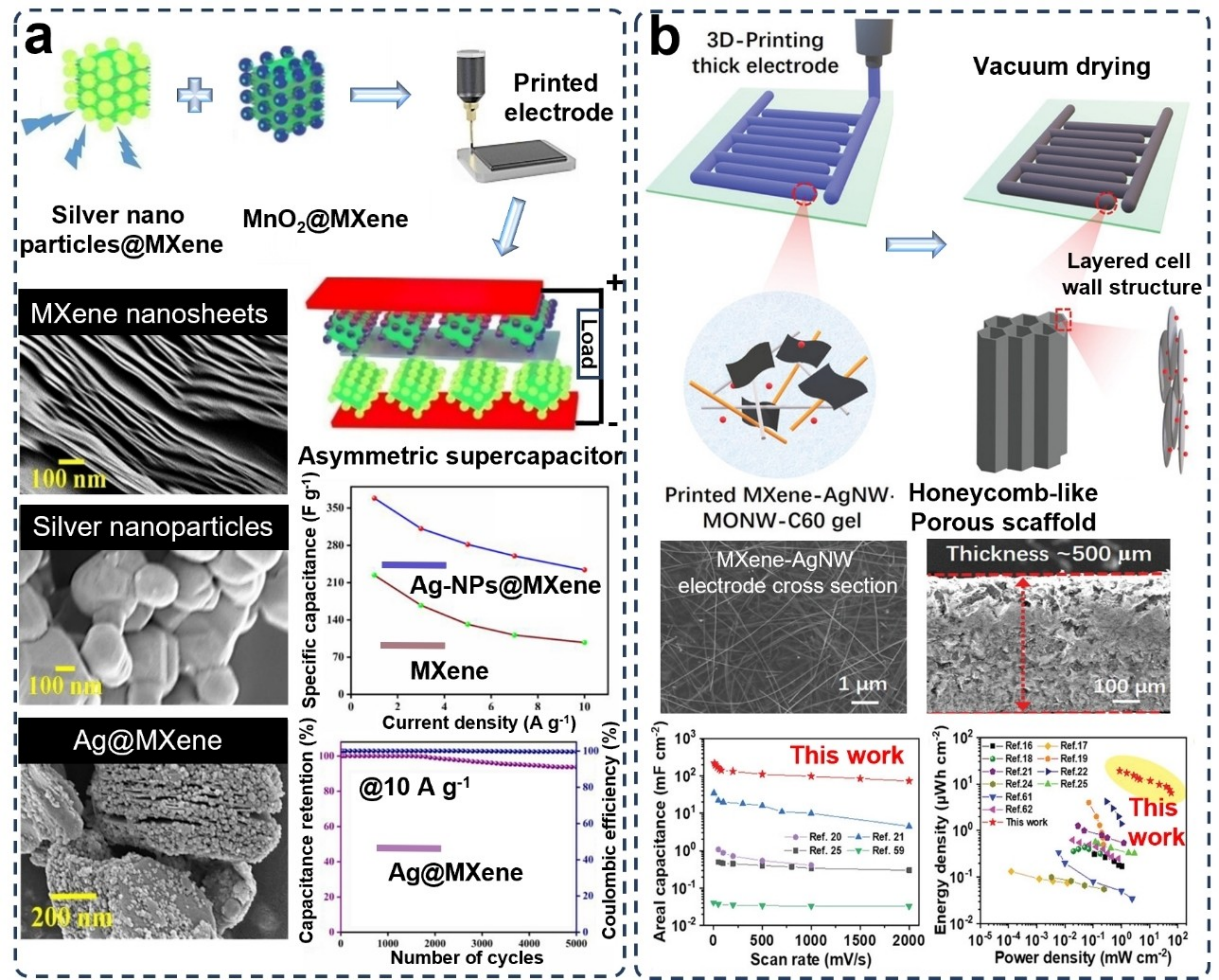
MXene/Metal electrodes for supercapacitors prepared via 3D printing. (a) MXene/silver (Ag) nanoparticles supercapacitor fabricated by DIW 3D printing. The SEM images illustrate the microstructures of MXene nanosheets, silver (Ag) nanoparticles and the Ag@MXene electrode. Graphics show the corresponding electrochemical performance. Reproduced from Ref. [84] with permission (2023) from Elsevier. (b) 3D printing of MXene/Ag nanowire supercapacitor via a DIW printing method. Reproduced from Ref. [86] with permission (2020) from Wiley‐VCH. The SEM images show the micro features of MXene‐Ag nanowires electrodes. Graphics illustrate high capacitance and energy density for this electrode.

Ag has been widely used in electrodes of energy devices for their excellent conductivity and electrochemical activities. To boost the redox reactions and suppress the agglomeration of MXene nanosheets in 3D printing, Ag nanoparticles have been added into the suspensions to form a 0D/2D hybrid ink for DIW printing of supercapacitor electrode shown in Figure 6a.^[84]^ The 3D printed Ag‐NPs@Ti_3_C_2_T electrodes for a symmetric supercapacitor reached a specific capacitance of 368.56 F g^−1^ at 1 A g^−1^ and a minimum specific capacitance of 233.84 F g^−1^ at 10 A g^−1^. In a 3D printed porous and hierarchical potassium‐ion layer double hydroxide (LDH) capacitor, a composite of nickel, cobalt, aluminum, and silver nano waves (NiCoAl−Ag) was incorporated with Ti_3_C_2_T_*x*_ to form a composite electrode with lower cost and longer lifespan.^[85]^ This MXene/metallic compound composite electrode enhanced the ion diffusion and mass transport of large potassium ions within the electrodes, leading to a specific capacitance around 55 mF cm^−2^ at a current density of 1 mA cm^−2^, and high cyclic stability of 80 % capacity retention after 10000 cycles. More importantly, the NiCoAl−Ag@Ti_3_C_2_T_*x*_ MXene ink exhibited optimal rheological properties for the 3D printing of electrodes. Multi‐metal intermetallic compounds could introduce multiple active atoms into electrodes for booting the electrochemical performance. Moreover, combining the 3D printing technique with other processes, such as freeze drying, can help the production of thick and porous MXene‐metal‐based supercapacitor electrodes. By printing the Ti_3_C_2_T_*x*_ MXene, fullerene, and manganese dioxide and silver nanowires into thick interdigitated electrode by using the DIW 3D printing method and the creation of a honeycomb‐like porous structure by utilizing a freezing drying procedure (Figure 6b), a MXene‐metal based electrode achieved an areal capacitance of 216.2 mF cm^−2^ at 10 mV s^−1^ and an energy density of 19.2 μWh cm^−2^.^[86]^

Through the study of MXene‐metal composite electrodes, it is clear the addition of highly conductive metallic materials improves the electrochemical performance of MXene‐based electrodes and enhances the reliability and stability of electrodes, due to the added metallic compounds could improve the rheological properties and avoid the agglomeration of the 2D materials during the preparation and printing of the inks, especially for preparing thick and porous electrodes. To expand this class of MXene‐based composite electrodes to practical, however, further efforts on further optimizing the types and content of the metallic materials, especially considering the compatibility of various components and the printability of high‐concentration inks for high‐load electrodes, are needed.

### 3.3 3D Printing MXene‐Metal Oxide Composite Electrodes

Metal oxides are very common electrodes materials and have been extensively used in various energy devices as active materials.^[87]^ Some typical transition metal oxides, such as the oxides of Mo, Mn, Co, Fe, Ni, Ti, Ru, etc., have been demonstrated to be promising materials for supercapacitor electrodes.^[88]^

**Figure 7:**
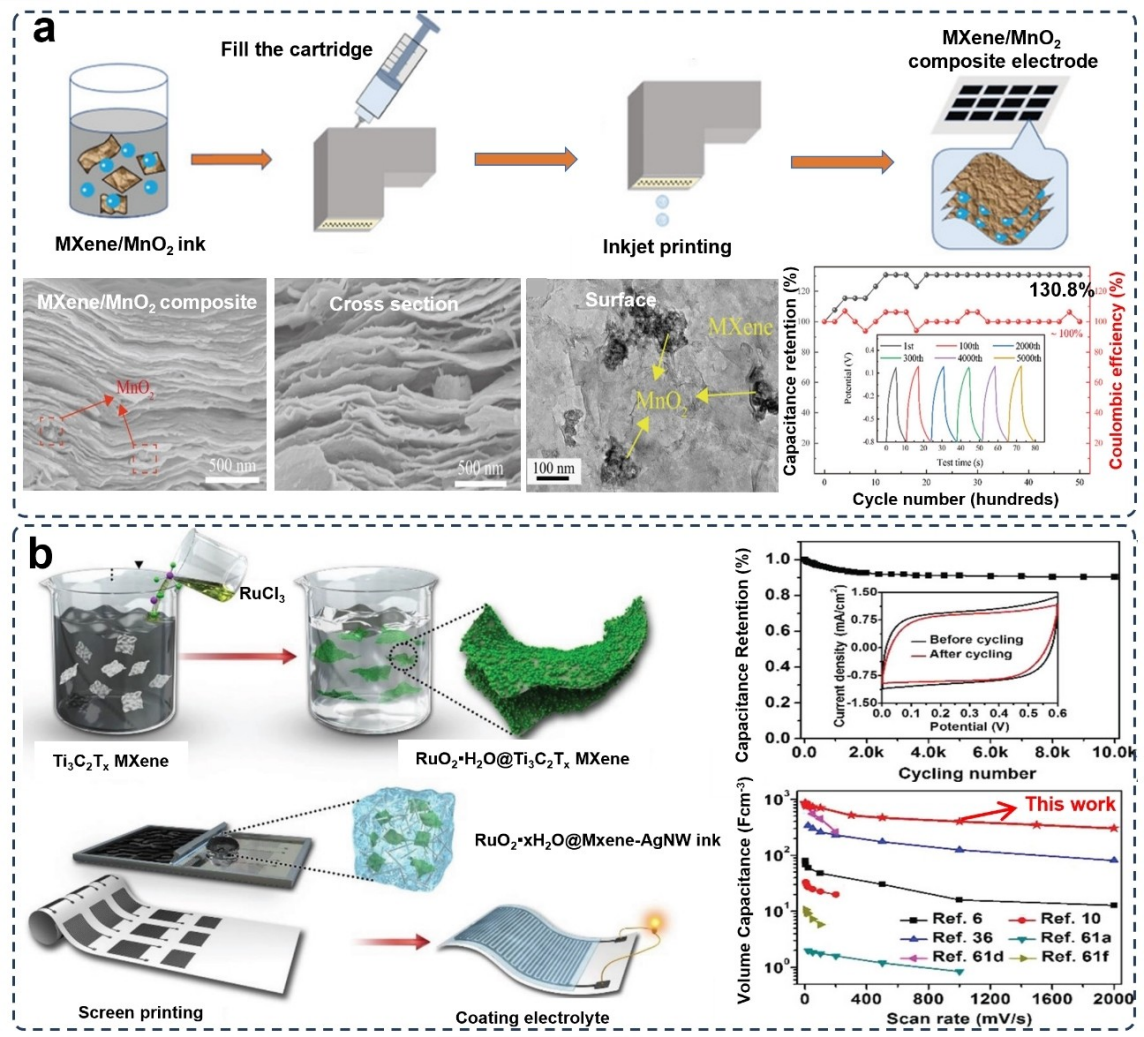
3D printing MXene/metal oxide composite electrodes for supercapacitors. (a) 3D printing of MXene/MnO_2_ electrodes via IJP printing method. SEM images show microstructures of the MXene/MnO_2_ electrode from cross section view and surface view, respectively. Stabile capacitance retention can be seen in the next graphic. Reproduced from Ref. [90] with permission (2021) from Wiley‐VCH. (b) The fabricating procedure of MXene/RuO_2_ 3D printing electrodes for high volume capacitance and stable capacitance retention which could be seen in the following graphics. Reproduced from Ref. [91] with permission (2019) from Wiley‐VCH.

In supercapacitors, the reversible reactions of metal oxides at the electrode interface during the charge and discharge processes can store more cations and thereby enhance the energy density.^[89]^ For example, a MXene/manganese dioxide (MnO_2_) composite electrode demonstrates highly efficient electron transport for reaching excellent overall electrochemical performance, where the MnO_2_ additive effectively prevents the MXene nanosheets from re‐stacking,^[90]^ and contributes to enhancing the effective electrochemical areas between the electrodes and the electrolyte. By using the IJP 3D printing method, this low‐cost film MXene/MnO_2_ composite electrode was fabricated (Figure 7a), which exhibited a remarkable conductivity of 3,400 S m^−1^, an extraordinary capacitance of 312 F cm^−3^, and 130.8 % capacitance retention after 5000 charge/discharge cycles. In an asymmetric supercapacitor composed of MXene/Ag nanoparticle composite anode and MXene/MnO_2_ composite cathode, a maximum energy density of 38.16 Wh kg^−1^ reached at a power density of 800 W kg^−1^, and a capacitance retention of 91.27 % after 5,000 cycles recorded.^[84]^ Decorated by ruthenium dioxide (RuO_2_) nanoparticles, a high‐performing fully printable MXene‐based electrode with micrometer‐scale resolution was designed (Figure 7b). In this work, silver nanowires (AgNWs) were added to guarantee the rheological property of the printing ink and provide conductive structures for the improvement of charge transport. This AgNWs‐doped RuO_2_/MXene micro‐supercapacitor finally performed a volumetric capacitance of 864.2 F cm^−3^ at 1 mV s^−1^, a capacitance retention of 90 % after 10000 cycles, a power density of 48.5 W cm^−3^.^[91]^

The issue of sedimentation and phase separation in metal oxide 3D printing inks has been a significant factor affecting their applications in 3D printing processes. In fact, the solution to the self‐restacking problem of MXene layers is not only limited to the addition of metallic compounds, but also including metal oxides and other types of materials. Metal oxides such as RuO_2_ and MnO_2_ can also form stable interactions with MXene. This interaction can be attributed to the strong chemical interaction between the hydrous metal oxides and the oxygen‐containing functional groups on the MXene surface, or the van der Waals forces between MXene and these metal oxide nanoparticles. The strong interfacial bonding between the materials enables the composite to stably exist in an aqueous environment, and further provides favorable conditions for the preparation of homogeneous 3D printable inks. As a consequence, the MXene/metal oxide composite ink not only can provide high capacitance, excellent capacitance stability, and strong mechanical properties for supercapacitor electrodes, but can also provide excellent uniformity for 3D printing inks. However, control of the viscosity of the printing ink cannot be ignored in 3D printing processing. A balanced ratio between the solid phase and the aqueous phase determines proper viscosity performance of the ink. This requires a large number of experiments for the 3D deposition molding of printing inks with different concentrations to obtain the best solid‐liquid ratio for a reliable effect. After the ratio is settled, with a proper control of printing ink viscosity, this strategy can provide better printing effects and a wider range of applications in the field of MXene 3D printed electrodes.

### 3.4 3D Printing MXene‐Carbon Composite Electrodes

Carbon‐based materials have been serving as both advanced structural materials for mechanical components and active materials in sustainable energy devices, for their exceptional mechanical properties and outstanding electric and electrochemical performances.^[92]^ For these unique properties, carbon‐based materials, such as emerging carbon nanomaterials (carbon dots, carbon nanotubes, and graphene), carbon fibers, and activated carbon materials, have been widely used in the application of supercapacitors, which needs superior electrochemical properties and structural stability.[^93^, ^94^] For example, as a typical 1D nanostructure, carbon nanotubes (CNTs), processing superior carrier mobility and mechanical properties can be fabricated into electrode via a DIW 3D printing technique, which offered a capacitance of 102 F g^−1^ at 2 A g^−1^ and retained 90 % of initial capacity after 7,500 cycles at 10 Ag^−1^.[^95^, ^96^, ^97^] Similarly, 2D graphene and graphene oxides also showed promising electrochemical performance as supercapacitor electrode. However, when used in aqueous inks for 3D printing, the hydrophobic property of graphene usually results in poor dispersion of the slurry. The combination of MXene with carbon‐based materials provides the opportunity to maximize the merits of both materials but overcome some disadvantages.

**Figure 8:**
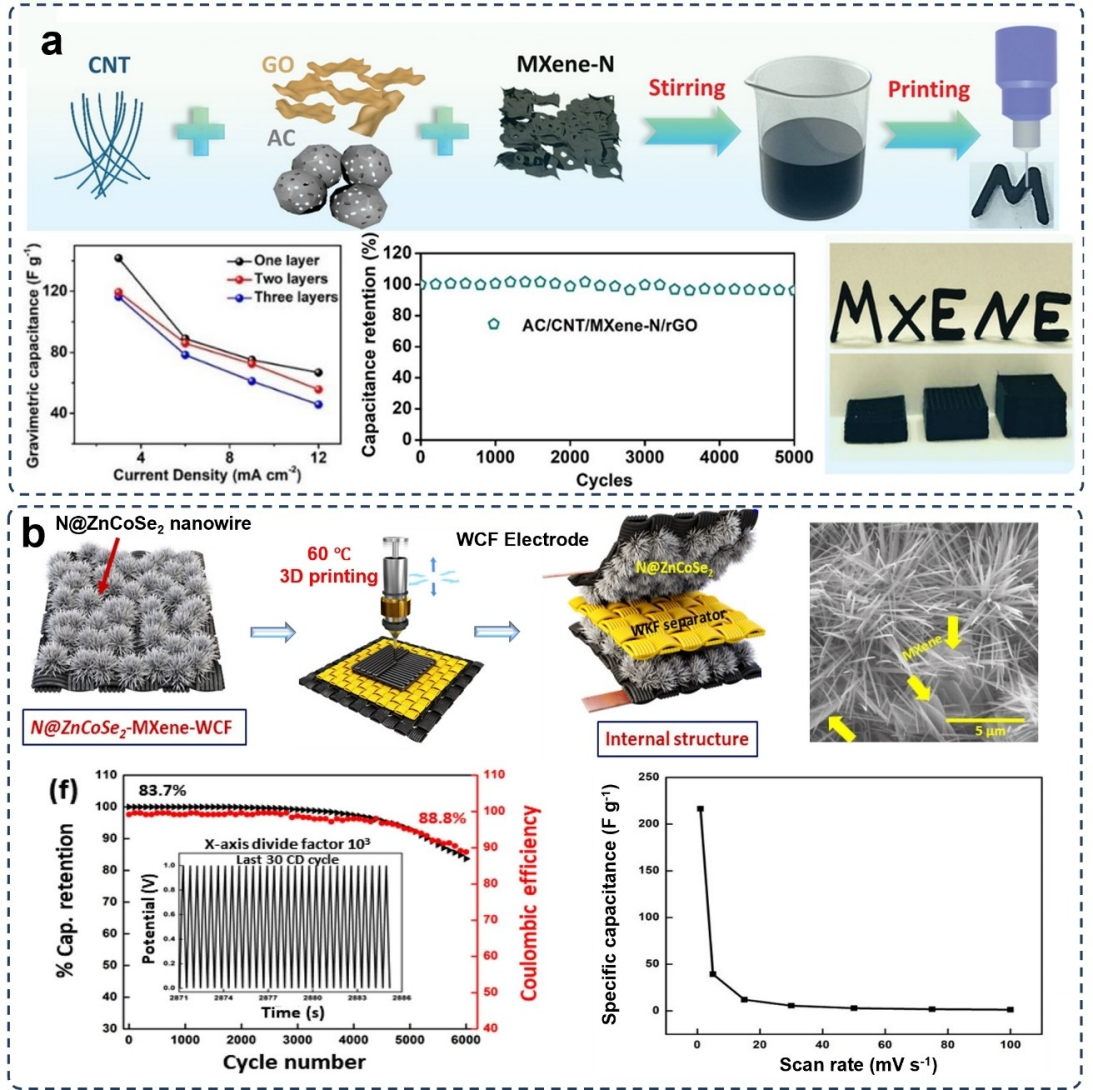
3D printing MXene‐carbon composite electrodes for supercapacitors. (a) Fabricating procedure for MXene/CNT electrode via DIW 3D printing. Graphics illustrate the high gravimetric capacitance and stabile cyclic capacitance of this device. Reproduced from Ref. [98] with permission (2019) from Wiley‐VCH. (b) FDM 3D printing of MXene/Carbon fiber (CF) composite electrodes. Reproduced from Ref. [101] with permission (2023) from American Chemical Society. The fabricating procedure and SEM images show the featured microstructure of this MXene‐Carbon fiber (CF) electrode. Graphics show the corresponding capacitance performance and capacitance retention of this electrode.

To improve the electrical properties of the MXene‐based ink, a binding‐free MXene/carbon nanotube (CNT) composite supercapacitor electrode was fabricated by using the DIW 3D printing technology. The combined 1D/2D nanostructures produced the inks with satisfactory rheological properties for 3D printing. The 3D printing MXene/CNT ink presented remarkable viscosity properties for DIW 3D printing, and the printed supercapacitor showed a maximum gravimetric capacitance of 66.7 F g^−1^ at a current density of 12 mA cm^−2^, and a capacitance retention of 96.2 % after 5000 cycles. (Figure 8a).^[98]^ It has been reported that the addition of binder into the MXene/CNT composite could further improve the printing performance of the inks. A porous MXene/CNT/chitosan composite electrode was obtained by adding the chitosan into the DIW 3D printing ink, which enhanced the rheological properties for performing the 3D printing process.^[99]^

Carbon fiber (CF) materials have been found the capability to provide ample energy storage and delivery capacity with very high mechanical strength.^[100]^ A MXene/CF electrode was fabricated (Figure 8b) by using the FDM 3D printing, where the CF was surface‐modified by synthesized Zn−Co selenide nanowires in the presence of MXenes.^[101]^ This electrode provided the supercapacitor with an excellent specific capacitance of 19.36 F g^−1^, and a capacitance retention of 83.7 % after 6000 cycles. While it is evident that 3D printed carbon composite electrodes can meet the demands of complex structures and offer high capacitance, the electrodes fabricated through this method still remain challenges. As 3D printing enables the control of electrode thickness to regulate volumetric capacitance and energy density. However, most electrodes limit ion diffusion under high mass loading, making them less competitive compared to traditional techniques such as evaporation, electrochemical deposition, and sputtering.^[102]^ Therefore, efforts should be directed towards researching suitable viscosity and porosity‐modified printable inks to achieve efficient conductive pathways.

### 3.5 3D Printing MXene‐Polymer Composite Electrodes

Conducting polymers have served as an intermediary material in batteries and supercapacitors to bind the inorganic electrode materials for better conductivity and structural integrity and fixity.^[103]^ A significant amount of conducting polymers is utilized in energy storage devices, such as polyaniline,^[104]^ polypyrrole,^[105]^ poly(3,4ethylenedioxythiophene):polystyrene sulfonate (PEDOT : PSS), etc. Polyaniline and polypyrrole were reported in the preparation of graphene‐based 3D printing inks to increase the rheological properties of the slurry.[^106^, ^107^] The PEDOT : PSS, is known for its adjustable electrical properties, unique redox properties, remarkable structure stability, reliable biocompatibility, and favorable water solubility.[^108^, ^109^, ^110^, ^111^] Due to the unique properties of PEDOT : PSS, it has been regarded as a promising additive for 3D printing inks. Apart from these conducting polymers, some polymers with inferior conductivity, such as polylactic acid (PLA) and acrylonitrile butadiene styrene (ABS), could also be used in electrode printing as scaffolds. These polymers can be combined with MXene materials for the preparation of 3D printing materials in the form of either filaments or inks.

A MXene‐decorated polymer‐based electrode for supercapacitor was prepared by FDM printing. In this procedure, a filament of PLA/TPU/carbon black (CB) was obtained via melting extrusion, and then PLA/TPU/CB electrode was 3D printed, and finally the polymer‐based electrode was solvent activated for MXene decoration on the surface.^[112]^ This conductive polymer‐based electrodes delivered a high capacitance of 20 mF cm^−2^ at 0.1 mA cm^−2^ and 95 % retention of capacity after 10,000 cycles. In this structure, the TPU/PLA polymers provide sufficient mechanical strength and toughness, the addition of carbon black enhances the electric conductivity and the capacitance of the electrode, and the addition of MXene much enhances the electrochemical performance of the electrode by providing the high conductivity and the pseudocapacitive charge storage on the nanosheets.

**Figure 9:**
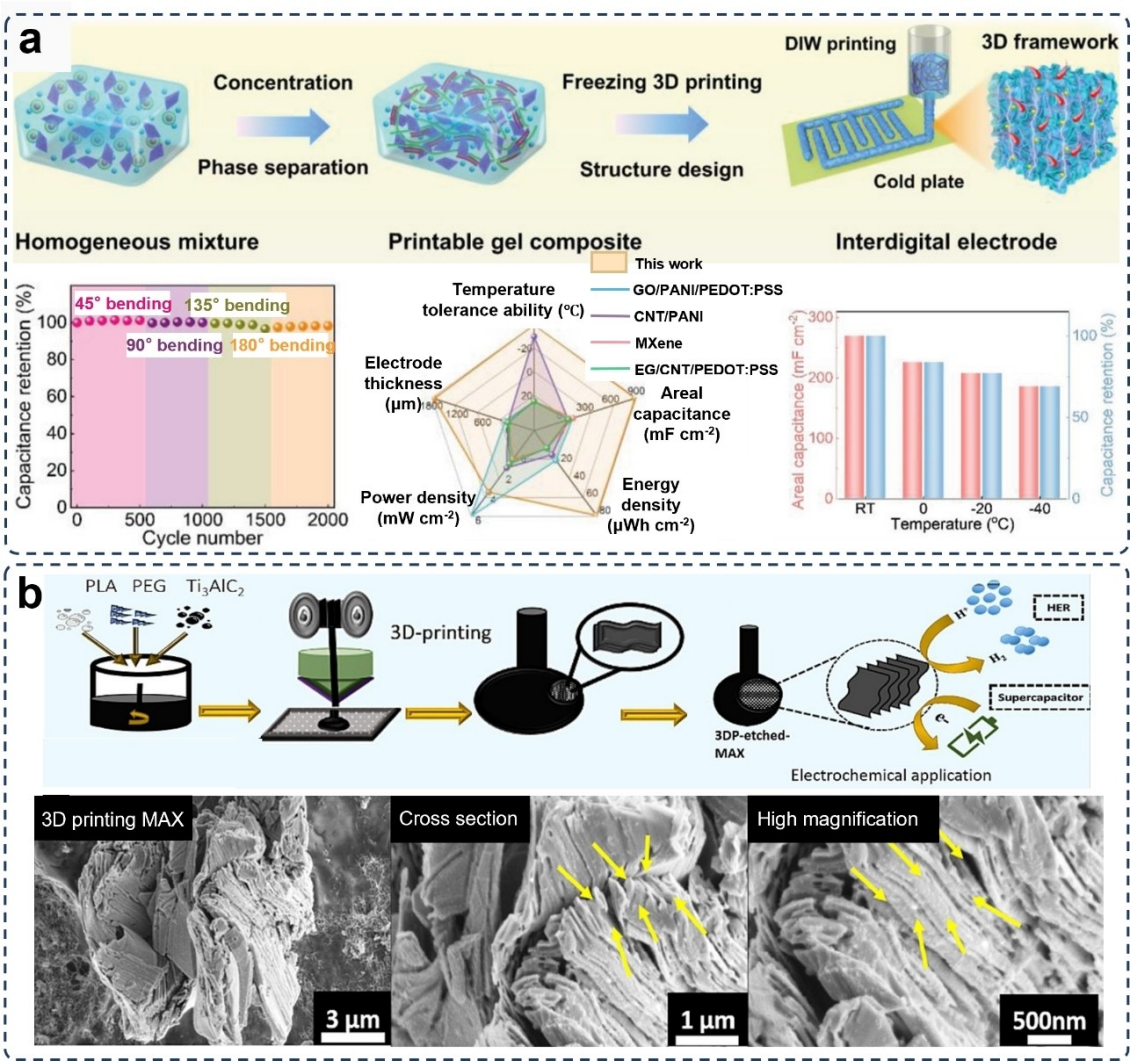
3D printing MXene‐polymer composite electrodes. (a) Preparation and DIW 3D printing of MXene/PEDOT : PSS composite electrodes and the capacity under temperature variations and deformations. Reproduced from Ref. [111] with permission (2023) from Wiley‐VCH. (b) In‐situ formation of MXene by integrating FDM 3D printing of MAX (Ti_3_AlC_2_)/polylactic acid (PLA) filament with a chronoamperometry etching for preparing 3DP‐etched‐MAX electrodes. SEM images show microstructure of the electrodes from different views Reproduced under terms of the CC‐BY license of Elsevier.^[114]^ Copyright 2024.

As plenty of functional groups existing on the surfaces and edges of MXenes, chemical bonds can be formed between these functional groups and the polyaniline and polypyrrole of the polymers to form uniform and stable networks.^[113]^ An eco‐friendly DIW 3D printing gel ink for MXene/PEDOT : PSS composite electrode was designed by mixing an aqueous PEDOT : PSS solution with the MXene nanosheets in an ethylene glycol (EG)/water solvent (Figure 9a).^[111]^ This gel ink facilitates the printing of 2D planar patterns with high geometric accuracy and exceptional shape fidelity benefiting from the functional groups of MXene the connection of connect with the neighboring PEDOT. Contributed by the optimized the ions and electrons transport kinetics induced by the interconnected bonding networks in the printed electrode, a larger areal capacitance of 889 mF cm^−2^, exceptional low‐temperature resistance with a capacitance retention of 85 % at −20 °C, and remarkable deformation tolerance under a 180° bending deformation.

As the preparation of MXenes, such as Ti_3_C_2_T_x_, usually needs the etching by HF. Very recently, an in‐situ MXene formed electrode was prepared by integrating FDM 3D printing of MAX (Ti_3_AlC_2_)/polylactic acid (PLA) filament with chronoamperometry etching stepwise in 9 M HCl and then 4 M NaOH (3DP‐etched‐MAX electrodes) (Figure 9b).^[114]^ This 3DP‐etched‐MAX electrode could be easily used as free‐standing working electrodes in hydrogen evolution reaction (HER) and supercapacitor applications without the need of current collectors.

It is very clear that the 3D‐printed MXene/polymer electrodes have some attractive advantages. (i) The 3D‐printed MXene/polymer electrodes usually present strong mechanical properties contributed by the interconnected bonds and networks formed between MXene nanosheets and macromolecules, which significantly improves the electrode stability and reduces the mechanical damage during processing and usage. (ii) The 3D‐printed MXene/polymer electrodes effectively take the advantages of both the 2D features of MXenes and the viscoelastic characteristics of the polymer precursors, which allows the preparation of homogeneous filaments and inks for 3D printing for the construction of complex structures. (iii) The easy moldability of polymers provides excellent flexibility to the 3D‐printed MXene/polymer electrodes, which expands their applications in wearable and portable electronic devices. (iv) The 3D‐printed MXene/polymer electrodes can possess desired ion/charge transfer properties via the choice of proper polymer materials. While this class of 3D printed composite electrodes need further efforts in solving the challenges in curing, environmental durability, wear, and friction resistance, etc., the 3D‐printed MXene/polymer electrodes offer a highly effective approach for the manufacturing of flexible electrodes of wearable and portable devices.

## 4 Summary and Outlook

This review provides an overview of 3D‐printed MXene‐based electrodes for supercapacitors/micro‐supercapacitors. Firstly, the commonly used 3D printing technologies for the printing of electrodes, including FDM, DLP, DIW, and IJP, were briefly introduced. Then, according to the starting materials for 3D printing, the printed electrodes for supercapacitors and their electrochemical performances were critically reviewed into five major categories: all‐MXene electrodes, MXene/metallic material composite electrodes, MXene/metal oxide composite electrodes, MXene/carbon material electrodes, and MXene/polymer composite electrodes. It is very clear that the combination of MXene materials with various additives provides great promising potentials in enhancing the performance of supercapacitors and the possible large‐scale production with customized characteristics. Although notable achievements have been made in 3D‐printed MXene‐based electrodes for supercapacitors, some grand challenges have to be addressed before pushing this type of technologies and devices into real‐world applications.

- Nanoengineering of MXenes for high load electrodes: it is out of the question that MXene materials possess salient properties for outperforming some other materials to be promising electrode materials for sustainable energy applications. However, the all‐MXene printing needs to address the restack issue during ink or filament preparation, especially for the preparation of high concentration inks for high load electrodes desired in practical applications. It has been revealed that the dimension and thickness of MXene sheets could affect the ink and printing properties of all‐MXene electrodes. Compared with micro‐sized MXene flakes, the nano‐sized flakes offer better ionic conductivity, but thicker flakes tend to have lower conformability and exhibit reduced sticking, due to decreased inter‐particle contact. By optimizing the size and the thickness of the MXene flasks, the high concentration all‐MXene inks could be modulated for the printing of high‐load electrodes. As electrostatic effect is one critical factor for long‐term stable suspensions, the tailoring of surface groups with high charges and the compositions of MXenes can induce highly charged surfaces for maintaining the desired stability and rheological properties for high‐load electrode printing. Besides engineering the MXene structures, the choice of solvents is another key factor to impose proper stacking behaviors to reach high concentration printing capability. While water and the polar organic solvents tend to promote a strong interaction between the MXene sheets, the non‐polar or low polarity solvents can weaken the interaction and provide better dispersibility of MXene sheets. Therefore, to prepare high concentration inks for the 3D printing of high load electrodes, nanosize and thickness engineering, surface functional group modulation, solvent selection, etc. could be approachable strategies.
- Heterostructuring and compositing of 3D printing MXene electrodes: It has been deemed to be an effective strategy to combine MXene materials with other materials to form heterostructures or composites for enhancing the energy storage properties and printing capability of MXene‐based electrodes. As we described in this review, diverse materials, including metals, metal oxides, carbon materials, and polymers, can be chosen as the second material or additive for the 3D printing of MXene‐based electrodes. According to the detailed case, proper MXene‐based heterostructures or composites could significantly enhance the loading amount and rheological properties of the inks, improve the electrochemical performance and expand the application areas of the MXene‐based materials.
- Materials compatibility in filament and ink preparation and 3D printing: For multi‐component printing filaments and inks, the compatibility of the constitutional materials determines the efficiency and accuracy of the 3D printed structures and the electrochemical performance of the electrodes. For the starting materials with poor compatibility can lead to severe aggregation and segregation of the constituents in the filaments or inks and then result in uneven distribution of the components/active materials in both the precursors and the printed electrodes. The inhomogeneity of the filaments or inks is also the major reason in nozzle blockage during printing and inferior electrode performance during service, such as low damage resistance, low electrical conductivity, and diminishing capacitance. Specifically, for the 2D materials like MXene, the restacking issue is also very significant, which also impacts the uniformity of the printing filaments or inks. In the selection of second‐phase or heterostructing materials for MXene‐based 3D printing, the factors that can enhance the compatibility of materials and then the rheological properties and the homogeneity of the filament/ink should be taken into consideration, such as attractive surface charges, matchable dimensionality and sizes, compatible chemical affinity, and proper wettability, etc. Besides considering the intrinsic properties of the constitutional materials, the addition of cross‐link reagents, which can provide interconnected bonds or interactions among the components, is also an effective solution to solving the compatibility issue. Furthermore, the 2D feature of MXenes also allows the modulation of the surface functional groups, which is also a tangible approach to enhance the electrostatic interactions for improving the compatibility between MXenes and other materials.
- Precision and accuracy of 3D printing: the precision control in the printing size, shape, and location is crucial for ensuring the accurate manufacturing of desired electrodes, particularly in the construction of micro‐sized electrodes for portable and wearable devices. It is a real challenge to print electrodes with sizes downwards micrometers and even nanomaterials, which, however, are highly expectable with the development of microelectronic devices. The reach of high precise and accuracy in microelectronic printing need dedicating designs in not only high‐resolution 3D printing machines but also perfectly prepared filaments or inks to fit with the micro‐sized nozzles. laser or photo‐induced 3D printing techniques offer high precision printing at a micrometre or even nanometre scale, particularly assisting with applied external fields, such as magnetic and electric fields. However, the limit in suitable photo‐curing resins induces additional difficulty in MXene‐based ink design and preparation. Thus, there is yet a long way to go to reach commercial applications of high‐resolution 3D printing of MXene‐based electrodes with large‐scale printing capacity and affordable costs.
- Optimization of printing parameters: The quality and productivity of 3D printing are governed by the setting of printing parameters, such as printing method, printing speed, extrusion pressure value, layer thickness, nozzle diameter, platform and print head temperature, supporting material and structure, and so on. For example, in DIW 3D printing, a faster printing rate can result in the extrusion of materials with a smaller diameter than the printing nozzle, leading to the construction of inaccurate final structure. During the printing process, the shinning behaviour of the inks could also result in the loss of precision and accuracy of 3D printing. In the printing of micro‐electrodes, the fluctuation of extrusion pressure can increase the homogeneity of the printing. Therefore, optimizing the printing parameters is very important to achieve high precise and accurate 3D printing.
- Scale production: For real‐world applications, the scaling production capability is essential. According to current progress, the scale production via 3D printing is yet limited by both the large‐scale preparation of long‐term stable inks and the 3D printing devices with rapid extrusion and curing techniques. Therefore, it is of significant importance to upgrade the hardware to enable rapid 3D printing and simultaneously high accuracy. Meanwhile, the sustainable synthesis of low‐cost and high‐quality MXene materials at scaling production is the first issue to be solved. The use of hazardous chemicals, such as HF, should be avoided. Thus, large‐scale 3D printing to meet the requirements of industrial scale production needs further contributions from mechanical engineers, material scientists, and chemists.

Therefore, 3D printing allows for the fabrication of electrodes with complex geometrical shapes, maximized surface areas, and optimized ion transport pathways, thereby enhancing the energy storage capacity of capacitors. Its flexible design capabilities can meet the demands for personalized customization and enable the rapid production and tests of different electrode prototypes. The employment of 3D printing techniques endorses the fabrication of various electrodes for energy devices at different scales. The 3D‐printed electrodes based on MXene materials offer a tangible option for high‐performance supercapacitors and micro‐supercapacitors which have the requirements in high‐resolution, complex shape, flexibility, and temperature resistance. If the challenges in the sustainable and scalable synthesis of MXenes and the bulk preparation of highly stable and dispersed MXene‐based inks and filaments for high‐load 3D printing could be solved, the combination of 3D‐printing technology with MXene‐based materials will enlighten the practical applications of 3D printed MXene‐based electrodes in portable and wearable electronic devices, energy storage devices, renewable energy technologies, hybrid vehicle energy systems, and lightweight aerospace equipment. Even though some challenges yet exist in the 3D printing of MXene‐based electrodes, this intelligent combination will surely unlock a promising potential in real‐world microelectronic and portable devices and expand horizon of sustainable energy storage technologies.

## Conflict of Interests

The authors declare no conflict of interest.

## Biographical Information

*Xudong Jiang received his bachelor's degree from Wuhan Institute of Technology in 2021. He is currently a postgraduate student under the supervision of Prof. Ziqi Sun, Prof. Ting Liao, and Dr. Juan Bai in School of Chemistry and physics at Queensland University of Technology, Australia. His research mainly focuses on the design and synthesis of 3D printing bioinspired multi‐functional materials*.

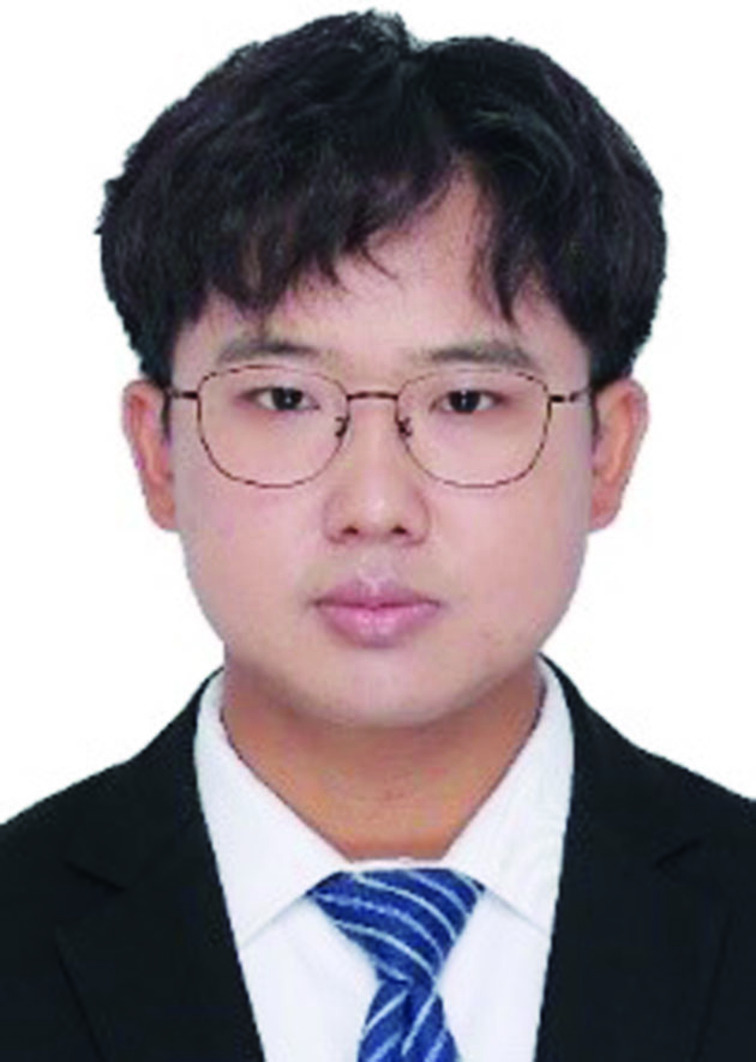

## Biographical Information

*Dr. Juan Bai is currently an ARC DECRA Fellow/Lecturer in the School of Chemistry and physics at Queensland University of Technology, Australia. She was a postdoctoral research fellow in the group of Prof. Ziqi Sun at Queensland University of Technology from 2020 to 2024. She received her PhD from Shannxi Normal University in 2019 under the supervision of Prof. Yu Chen and MS from Nanjing Normal University in 2015. Her research interest mainly focuses on the design and synthesis of functional nanostructured catalysts for electrochemistry and energy conversion devices*.

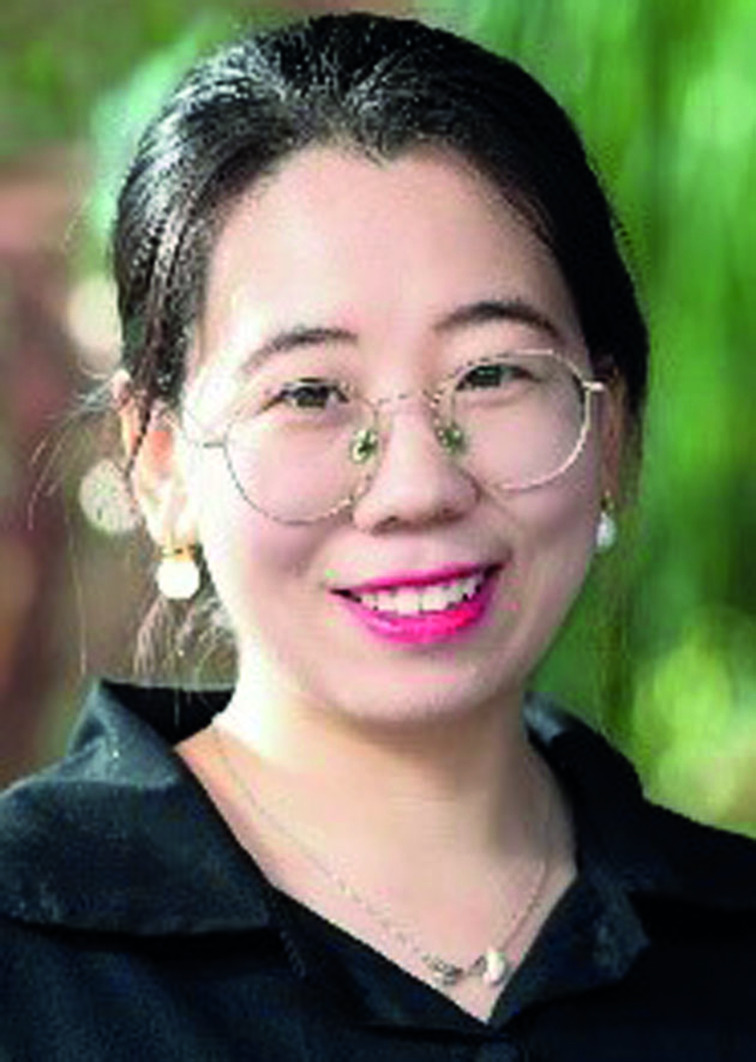

## Biographical Information

*Prof. Ting Liao is a full professor in the School of Mechanical, Medical, and Process Engineering, Queensland University of Technology, Australia. She received her PhD in 2009 from Institute of Metal Research, Chinese Academy of Sciences. Since then, she was awarded four prestigious fellowships and worked at National Institute for Materials Science, Japan, the University of Queensland and University of Wollongong, Australia. In 2017, she joined Queensland University of Technology, as a Senior Lecturer and the ARC Future Fellow. Prof. Liao is interested on low dimensional materials in diverse energy applications, including photovoltaics, catalysis, and energy storage*.

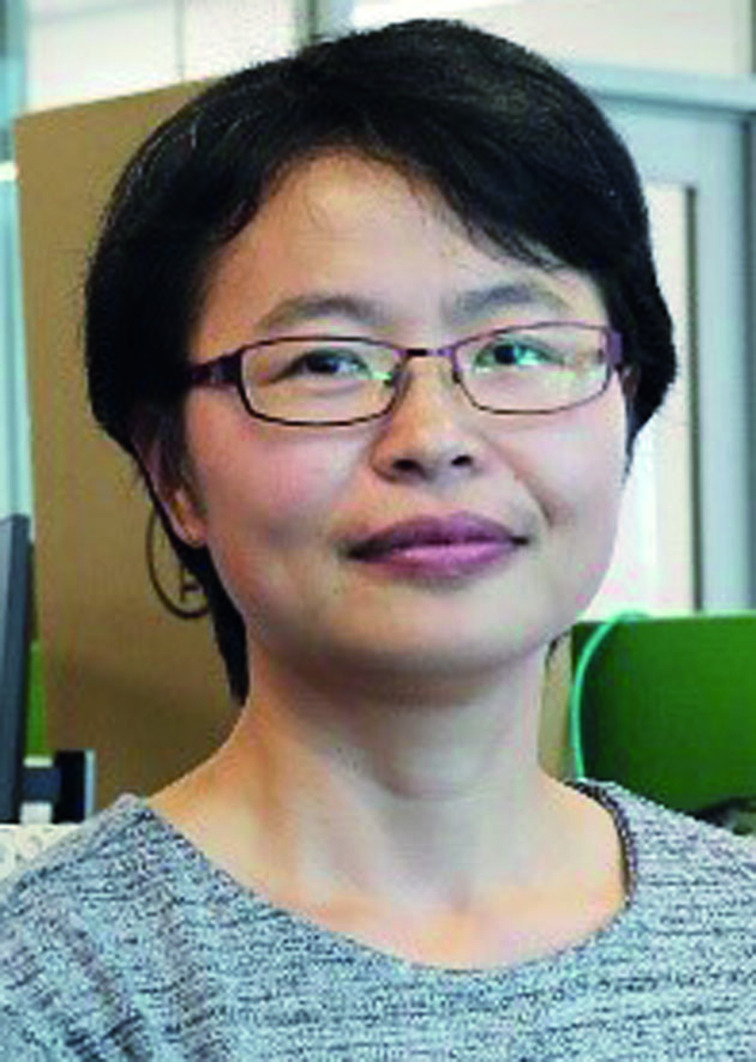

## Biographical Information

*Prof. Ziqi Sun is currently a full professor at the Queensland University of Technology, Australia. His research interest includes developing bioinspired smart nanomaterials and 2D metal oxide nanomaterials for sustainable energy and environmental applications, such as rechargeable batteries, oil‐water separations, and sustainable fuel generations. Ziqi received his PhD degree from Institute of Metal Research, Chinese Academy of Sciences in 2009. After one‐year experience as NIMS postdoctoral fellow (Japan), he joined University of Wollongong, Australia in 2010 and moved to QUT as a faculty member in 2015*.